# Autoimmune hemolytic anemia, a rare disease in newborns: a case report

**DOI:** 10.1097/MS9.0000000000000681

**Published:** 2023-04-18

**Authors:** Sandhaya Kukreja, Saadia Abu Baker, Sidhant Ochani, Sapna Lohana, Asifa Kalwar, Kainat Memon, Sadhna Kumari, Muhammad Faraz, Md. Al Hasibuzzaman

**Affiliations:** a Department of Medicine, Dow University Of Health and Sciences, Karachi; b Department of Medicine, Khairpur Medical College, Khairpur Mir’s; c Department of Medicine, Peoples University Of Health Sciences For Women, Nawabshah; d Department of Medicine, Shaheed Mohtarma Benazir Bhutto Medical University, Larkana, Pakistan; e Institute of Nutrition and Food Sciences, University of Dhaka, Dhaka, Bangladesh

**Keywords:** autoimmune hemolytic anemia, AIHA, cardiac defects, newborn, sepsis

## Abstract

Autoimmune hemolytic anemia (AIHA) is a type of hemolytic anemia in which autoantibodies attack the membrane antigens of red blood cells, causing cell rupture (lysis). Hemolysis stimulates compensatory RBC production by boosting erythropoietin levels; however, this response is often insufficient to restore normal hemoglobin blood levels, resulting in anemia. It is a rare disease, with an annual incidence of one case in every 80 000 live births. Infants of any age can be affected, though neonatal incidence is unusual. Here, the authors report a rare case of AIHA in the neonatal period with concomitant atrial septal defect, ventricular septal defect, and patent ductus arteriosus.

**Case presentation:**

A one-hour-old male neonate weighing 3 kg who was born at 38 weeks of gestation presented to the pediatric department with the complaint of respiratory distress. Examination revealed obvious respiratory distress with subcostal and intercostal recessions and a continuous grade 2 murmur at the left upper chest; the liver was palpable 1 cm below the right subcostal margin with a palpable splenic tip. Laboratory investigations were ordered, which showed hemoglobin was decreasing continuously and bilirubin was raised, suspecting AIHA. A positive blood culture, tachycardia, tachypnea, and a raised leukocyte count showed that the baby was in sepsis. The baby improved clinically, and the complete blood count showed improved Hb. Cardiac examination findings and a second-grade continuous murmur at the left upper chest were further investigated through echocardiography, which showed a grade 2 atrial septal defect, a muscular ventricular septal defect, and a patent ductus arteriosus.

**Clinical discussion:**

Childhood AIHA is a rare and underrated disease that differs from the adult form. The disease’s initial manifestation and subsequent course are both poorly understood. It affects mostly young children, and a high prevalence (21%) is found in infants. In some patients, there is a genetic predisposition to the development of this disease, and there is underlying immune deregulation in more than half of the cases, necessitating long-term homogeneous multidisciplinary follow-up. It is of two types, primary and secondary, and according to the study conducted in France, AIHA is associated not only with other autoimmune diseases but with some systemic diseases as well, like neurological, digestive, chromosomal abnormalities, and cardiac diseases, as in our case.

**Conclusion:**

There is a scarcity of data on clinical management and treatment strategies. More research should be done to know the environmental factors that can trigger the immune response against red blood cells. Moreover, a therapeutic trial is essential for a better outcome and helps prevent serious complications.

## Introduction

Highlights

- Acute hemolytic anemia may be preceded by an infection in infants.
- It can spontaneously remit or respond to corticosteroids, as in our case.
- There is a scarcity of data on clinical management and treatment strategies.
- Steroids are the first-line treatment, long-term use is not considered due to undesirable side effects.
- More research should be done to know the environmental factors that can trigger the immune response against red blood cells.

Autoimmune hemolytic anemia (AIHA) is a type of hemolytic anemia in which autoantibodies attack the membrane antigens of red blood cells (RBCs), causing cell rupture (lysis). Hemolysis stimulates compensatory RBC production by boosting erythropoietin levels; however, this response is often insufficient to restore normal hemoglobin (Hb) blood levels, resulting in anemia^1^. It is most typically caused by IgG autoantibodies aimed against RBC surface proteins, but IgM autoantibodies interacting with RBC polysaccharides can also cause it. It can be primary/idiopathic or secondary to infections (Epstein-Barr Virus, Cytomegalovirus, mycoplasma), drugs (penicillin, quinidine, and a-methyldopa), chronic autoimmune disorders (SLE and ulcerative colitis), and an underlying malignancy (chronic lymphocytic leukemia, nonHodgkin’s lymphoma). It can present with pallor, respiratory difficulty, jaundice, lethargy, cyanosis, abdominal pain, low-grade fever, dark urine (severe hemolysis), and hepatosplenomegaly. The majority of cases are acute and have a better prognosis, with spontaneous remission possible within 6 months. The remaining cases are chronic, making treatment more challenging. The treatment options available are exchange transfusion, plasmapheresis, steroids, intravenous immunoglobulin (IVIG), splenectomy, warming, and rituximab^2^. The direct antiglobulin test, which is a highly sensitive and relatively specific test, is primarily used for the diagnosis of AIHA. AIHA is classified as warm and cold type on the basis of the pattern of the direct antiglobulin test and on the immunochemical properties of the antibodies. One of the most important steps in the diagnosis of AIHA is to distinguish between AIHAs due to warm antibodies (wAIHA) and AIHAs due to cold antibodies since it affects the therapeutic approach. While corticosteroids are the mainstay of treatment for wAIHA, they are usually not effective for cold AIHA^3^. It is a rare disease, with an annual incidence of one case in every 80 000 live births. Infants of any age can be affected, though neonatal incidence is unusual^4^. Here, we report a rare case of AIHA in the neonatal period with concomitant atrial septal defect (ASD), ventricular septal defect (VSD), and patent ductus arteriosus (PDA). This case has been reported in line with Surgical CAse REport (SCARE) 2020 Guidelines^5^.

## Case presentation

### Chief complaint

A one-hour-old male neonate weighing 3 kg who was born at 38 weeks of gestation presented to the pediatric department with the complaint of respiratory distress.

### History of present illness

The baby had an immediate cry, vitamin K was given soon after birth, and there was no history of resuscitation after birth. On examination, he was pale, jaundiced up to the abdomen, and had bluish discoloration of the feet, but was fully conscious and alert. Baby’s vitals were: heart rate 124/min, respiratory rate 68/min, temperature 98F, random blood sugar 60 mg/dl, and saturation 98%. Apgar scores were seven and eight at 1 and 5 min, respectively. Anthropometric measurements were within the normal range. Further examination revealed obvious respiratory distress with subcostal and intercostal recessions and a continuous grade 2 murmur at the left upper chest; the liver was palpable 1 cm below the right subcostal margin with a palpable splenic tip.

### Birth history

He was the fourth product of a consanguineous marriage. An emergency C-section was performed because of the history of ruptured membranes and leakage. Antenatal history was not significant except that the mother was diagnosed with gestational diabetes mellitus during the fourth month of gestation, which was well controlled.

### Laboratory findings

To evaluate the examination findings, some laboratory investigations were ordered, which showed low Hb. For furthur evaluation of low Hb, investigations including serum bilirubin levels were ordered, as shown in Table 1.

**Table 1:** Laboratory findings pre post-transfusion

Labs	First Day (Pre-transfusion)	Third Day (Post-transfusion)
Hb	9.9 g/dl	12.1 g/dl
MCV	136.9 fl	85.6 fl
MCH	42.5 pg	33.9 pg
MCHC	31 g/dl	30 g/dl
TLC	39.5×10e9/l	10.3×10e9/l
Neutrophils	31%	50%
Lymphocytes	58%	44%
Platelets	215×10e9/l	135×10e9/l
PT	12.8 s	11.2 s
APTT	24.5 s	23.8 s
INR	1.22	1.1
T. Bili	13.9 mg/dl	12.9 mg/dl
D. Bili	1.5 mg/dl	2.7 mg/dl
In. Bili	12.4 mg/dl	10.2 mg/dl
CRP	–	mg/l

APTT, Activated Partial Thromboplastin Cloting Time; CRP, C-Reactive Protien; D. Billi, Direct Bilirubin; Hb, Hemoglobin; In. Billi, Indirect Bilirubin; INR, International Normalized Ratio; MCH, Mean Corpuscular Hemoglobin; MCHC, Mean Corpuscular Hemoglobin Cocentration; MCV, Mean Corpuscular Volume; PT, Prothrombin Time; T. Billi, Total Bilirubin; TLC, Total Leukocytes Count.

### Family history

Incompatibility occurs in newborns who have type A, B, or AB blood group and whose mothers’ blood group is O. In our case, the mother’s blood group was B negative and the baby’s blood group was O, which has no antigen to trigger an immune response. In the case of ABO incompatibility, it must have affected previous pregnancies, and should be less severe in this case but was not; this ruled out ABO and Rh incompatibility as a possible cause of jaundice. There was no family history of hereditary spherocytosis, sickle cell anemia, or thalassemia.

### Medications

Conservative management was started, and the baby was kept on CPAP, maintenance fluids, cefotaxime 150 mg IV BD, amikacin 25 mg IV BD, and double phototherapy with an eye pad was applied for 3 days.

### Diagnostic studies

His Hb was decreasing continuously and bilirubin was raised (Hb: 8.8 g/dl, TLC: 38×10 e9/l, T. Bili: 14.1 mg/dl, D. Bili: 2.1 mg/dl, and In. Bili: 12.0 mg/dl). Consequently, whole blood was arranged and an exchange transfusion was done on the second day of life. Post-transfusion complete blood count (CBC) and serum bilirubin levels are shown in Table 1. His peripheral blood smear revealed a dimorphic picture, anisocytosis, poikilocytosis, polychromasia, and nucleated RBCs, and the reticulocyte count was 16.60%; Continuously dropping Hb, increasing bilirubin along with the peripheral blood smear supported ongoing hemolysis. A Glucose 6PD enzyme assay was done, which was within the normal range, which excluded Glucose 6PD deficiency as a possible cause of hemolysis. Serum lactate dehydrogenase was raised (986 U/L), and the direct Coombs test was positive. On the fourth day of life, his Hb dropped back to 11 g/dl and serum bilirubin levels were increased; this suggested ongoing hemolysis. The direct Coombs test was positive again and prednisolone 2 mg/kg/day was started, suspecting warm AIHA. Belonging to a third world country, we tried to offer the minimum required investigations to reach a diagnosis and effectively treat the patient as the patient could not afford, preventing cost-burden. From the blood grouping test, we concluded that in the type of blood grouping pair (O negative baby and B negative mother), ABO incompatibility cannot occur. While other types of alloimmune hemolytic anemia are very rare and cannot cause hemolysis to such extent present in this baby.

### Follow-up

Investigations were repeated again 4 days after the steroid use which are shown in Table 2. Repetitively positive direct Coombs tests and improvements in Hb and bilirubin levels after the steroid use strongly supported the diagnosis of warm AIHA. On the first day of stay in the hospital, the CBC showed a raised TLC count of 39.5×10 10e9/l; it was raised in the subsequent CBCs too, and the baby had two fever spikes during the hospital stay. The urine culture revealed no growth, whereas the blood culture revealed the presence of micrococcus bacteria. A positive blood culture, tachycardia, tachypnea, and a raised leukocyte count showed that the baby was in sepsis. There was no history of autoimmune disease in the family, and the AIHA in this baby could be either idiopathic or secondary to infection as the baby was in sepsis.

**Table 2:** Laboratory findings after steroid use

Labs	After steroids
Hb	12.5 g/dl
MCV	81.4 fl
MCH	34.3 pg
MCHC	28 g/dl
TLC	10.7×10e9/l
Neutrophils	45%
Lymphocytes	34%
CRP	39.3 mg/l
Platelets	122×10e9/l
T. Bilirubin	3.3 mg/dL
D. Bilirubin	1.4 mg/dl
In. Bilirubin	1.9 mg/dl

CRP, C-Reactive Protien; D. Billi, Direct Bilirubin; Hb, Hemoglobin; In. Billi, Indirect Bilirubin; MCH, Mean Corpuscular Hemoglobin; MCHC, Mean Corpuscular Hemoglobin Cocentration; MCV, Mean Corpuscular Volume; T. Billi, Total Bilirubin; TLC, Total Leukocytes Count.

### Outcomes

The baby improved clinically, and the CBC showed improved Hb: 12.5 g/dl. The dose of steroid was tapered slowly, and the antibiotics were changed to tazobactam and piperacillin 110 mg/kg/day for 7 days, according to the culture and sensitivity reports.

### Review of systems

Cardiac examination findings and a second-grade continuous murmur at the left upper chest were further investigated through echocardiography, which showed grade 2 ASD, muscular VSD, and PDA. AIHA was associated with congenital cardiac anomalies in this baby and the baby was referred to the cardiology department for further evaluation, where he was asked to appear for follow-up after 1 month. Echocardiography was repeated which showed resolved ASD, VSD, and PDA, however, the patient had a mild patent foramen ovale, which was also subsequently resolved after 6 months of re-evaluations.

## Discussion

Childhood AIHA is a rare and underrated disease that differs from the adult form. The disease’s initial manifestation and subsequent course are both poorly understood. It affects mostly young children, and a high prevalence (21%) is found in infants. In some patients, there is a genetic predisposition to the development of this disease, and there is underlying immune deregulation in more than half of the cases, necessitating long-term homogeneous multidisciplinary follow-up. It is of two types, primary and secondary, and according to the study conducted in France, AIHA is associated not only with other autoimmune diseases but with some systemic diseases as well, like neurological, digestive, chromosomal abnormalities, and cardiac diseases, as in our case^6^.

The initial objectives of treatment are decreasing hemolysis, stabilizing Hb levels, and increasing the safety and tolerability of packed RBC transfusions, if needed. RBC transfusions should be given to patients with severe anemia to keep their Hb at a clinically tolerable level, at least until other therapeutic approaches are used to stop the hemolysis and the patient’s bone marrow compensate for the rapid RBC depletion. The recommendation for blood transfusion is mainly based on the severity of the hemolysis, the progression of the anemia and, more importantly, to the associated clinical findings. RBC transfusion may be required, especially in cases of life-threatening anemia, in patients with coronary artery disease who have an elevated risk of cardiac or cerebral events, and in symptomatic elderly individuals^7^–^12^. Glucocorticoids, the first-line of treatment, are normally administered orally as prednisone or prednisolone, though early intravenous methylprednisolone administration may be necessary depending on the clinical condition of the patient. Up to 80% of patients respond positively to steroids and usually show a response within 24–48 h after initiation. Steroids should be gradually taken off after Hb returns to normal over the course of about six months, as rapid tapering or abrupt withdrawal have been linked to disease relapse^13^. When the response to steroids in the acute context is poor, such as with persistent hemolysis requiring multiple transfusions 24–48 h after steroid treatment, we move on to the additional first-line treatment of IVIG. A mixed prospective and retrospective study by Flores^14^ reported a positive response to IVIG in 29 of 73 patients (39.7%), including 6 out of 11 children (54%), with two variables strongly associated with a good response: the presence of hepatomegaly with or without splenomegaly and a low pretreatment Hb. Second-line therapy must be introduced sooner due to the poor full remission rate of 58% after the first month of steroid therapy and the toxicity of these medications. Rituximab (anti-CD20 antibody), splenectomy, and immunosuppressive medications are among the 265 second-line choices^15^. However, there are no controlled studies or evidence-based data for the best treatment of childhood AIHA yet^11^,^16^.

## Conclusion

Thus, it is concluded that AIHA is a rare disease in neonates, and its etiology is uncertain in most of the cases. Acute hemolytic anemia may be preceded by an infection and spontaneously remits or responds to corticosteroids, as in our case. There is a scarcity of data on clinical management and treatment strategies. Although steroids are the first-line treatment, long-term use is not considered due to undesirable side effects. More research should be done to know the environmental factors that can trigger the immune response against red blood cells. Moreover, a therapeutic trial is essential for a better outcome and helps prevent serious complications. A lack of willingness to transfuse patients with AIHA due to ambiguity about the safety and efficacy of RBC units that are incompatible due to the presence of RBC autoantibody is one of the most frequent management errors, according to studies.^17^ Prior to transfusion in such patients, significant effort is frequently made to assess the indication and serological compatibility. However, on occasion, this could put patients who urgently need a red blood cell transfusion in danger. Not the compatibility test results, but rather the assessment of the patient’s need for transfusion, is the most crucial factor in transfusion in AIHA. The patient was treated successfully with the aforementioned treatment, proper follow-up was done, and management was done accordingly. On the last follow-up the patient was doing well.

## Ethical approval

Dow University of Health and Sciences. (IRB Reference Number: DUHS/22/177).

## Consent

Written informed consent was obtained from the patient for publication of this case report and accompanying images. A copy of the written consent is available for review by the Editor-in-Chief of this journal on request.

## Sources of funding

The author(s) received no financial support for the research, authorship, and/or publication of this article.

## Author contributions

S.K. and S.A.B.: data collection; K.M., M.F., and S.K.: literature review; S.K., S.A.B., S.O., S.L., and M.A.D.: review-editing; A.K. and S.O.: referencing and formatting. Manuscript was written by all authors.

## Conflicts of interest disclosure

The author(s) declared no potential conflicts of interest concerning the research, authorship, and/or publication of this article.

## Research registration unique identifying number (UIN)

Not Applicable.

## Guarantor

All authors accept full responsibility for the work and/or the conduct of the study, had access to the data, and controlled the decision to publish.

## Patients’ perspective

I am satisfied by the treatment and follow-up doctors’ went through managing my case and saving my child, May God bless you all.

## Provenance and peer review

Not commissioned, externally peer reviewed.
